# Carbon Nanofibers Based on Potassium Citrate/Polyacrylonitrile for Supercapacitors

**DOI:** 10.3390/membranes12030272

**Published:** 2022-02-27

**Authors:** Wang Zhang, Ludan Zhang, Junqiang Guo, Jeongyeon Lee, Liwei Lin, Guowang Diao

**Affiliations:** 1 School of Chemistry and Chemical Engineering, Yangzhou University, Yangzhou 225009, China; zhangwang@yzu.edu.cn (W.Z.); zhangludan01@163.com (L.Z.); guobyxg@hotmail.com (J.G.); 2 Department of Applied Bioengineering, Graduate School of Convergence Science and Technology, Seoul National University, Suwon-si 16229, Korea; 3 Institute of Textiles Clothing, Faculty of Applied Science and Textiles, The Hong Kong Polytechnic University, Hung Hom, Hong Kong SAR 999077, China; jaden-jy.lee@polyu.edu.hk

**Keywords:** nanofiber membrane, supercapacitor, electrospinning, potassium citrate

## Abstract

Wearable supercapacitors based on carbon materials have been emerging as an advanced technology for next-generation portable electronic devices with high performance. However, the application of these devices cannot be realized unless suitable flexible power sources are developed. Here, an effective electrospinning method was used to prepare the one-dimensional (1D) and nano-scale carbon fiber membrane based on potassium citrate/polyacrylonitrile (PAN), which exhibited potential applications in supercapacitors. The chemical and physical properties of carbon nanofibers were characterized by X-ray diffraction analysis, scanning electron microscopy, transmission electron microscopy, Raman spectroscopy, X-ray photoelectron spectroscopy, and the Brunnauer–Emmett–Teller method. The fabricated carbon nanofiber membrane illustrates a high specific capacitance of 404 F/g at a current density of 1 A/g. The good electrochemical properties could be attributed to the small diameter and large specific surface area, which promoted a high capacity.

## 1. Introduction

Energy has played an irreplaceable role in promoting the progress of civilization and human living standard [1]. With the rapid development of the economy and social changes, the shortage of resources and environmental problems caused by excessive consumption of fossil energy have attracted more attention. Thus, the development of clean energy has become extremely urgent [2]. In the process of clean energy development and utilization, how to effectively store the clean energy is a key strategy, which requires devices with high energy density and power density [3]. Facing this challenge, how to develop green, renewable, and efficient energy storage technology is the responsibility of many electrochemical researchers [4,5]. In order to meet the development of smaller and faster charging and discharging of portable and wearable electronic devices in modern optoelectronics, supercapacitors serve as convenient micro electrochemical energy storage devices that can provide a promising opportunity to overcome this problem [6].

As the overall performance of supercapacitor is closely related to the electrode materials, material science becomes the key to develop the new generation of energy storage devices [7]. One synthesis strategy is based on the manufacture of nano-sized porous carbon (<200 nm), which can provide a large specific surface area or a short diffusion path (<100 nm). Three-dimensional (3D) structures have abundant cavities, such as high-quality mesoporous interconnections [8], but such structures significantly reduce the effective ion transport distance. Several multi-step processes have been reported for making 3D porous carbon nanoparticles, but they all require expensive reagents [9]. Although two-dimensional (2D) layered nanomaterials exhibit high specific capacitance, the space required to increase the specific device energy and power varies greatly depending on the application objectives. There is another problem that cannot be ignored in 2D nano-layered materials. Although they have effective ion and electron transport speeds in the horizontal direction of 2D lamellar, there exist some obstacles in the ion or electron transport in the vertical direction, resulting in the difficult reaction between penetrate into the electrode and the realization of fast storage. Furthermore, the ideal capacitance of the nanofibers obtained by electrospinning is based on the unique 1D nanostructure, which enables the electron transport to be directional [10]. In terms of the unique 1D nanostructure, the electron transport length is greatly shortened, thus greatly enhancing the efficiency [11,12]. On top of that, the stable continuity and flexibility of the 1D nanofiber membrane are the prerequisite for the application of binder-free electrode materials.

It cannot be ignored that the introduction of adhesive components would greatly reduce the electrical conductivity of the active materials, even blocking the porous structures. Further, the most serious consequences are poor cyclic stability and lower multiplier performance. Adhesive-free electrode materials not only avoid a series of problems, but also simplify the complex electrode manufacturing process, exhibiting greatly practical application prospects. At present, a large number of researchers are pursuing in-depth studies on unbonded electrode materials, and electrospinning manufactured nanostructure materials have aroused more and more attention because of their simple preparation and strong plasticity [13,14,15]. Electrospinning as an adjustable process can design nanofibers with versatile structures for energy storage devices. It is primarily combined with features of CVD and hydrothermal methods to further tailor the structure and composition of nanofibers. Electrospinning advantages include (a) mass production of nanofibers with low cost, (b) facile fabrication of controllable nanofiber features in terms of morphology, structure, and composition, and (c) easy acquisition of 3D freestanding membranes [16,17,18,19]. The fusion of multiple components (carbon materials, [20], conductive polymers, [21], metal oxides [22], etc.) can be achieved through blending, the sizes of which can be effectively controlled through high-temperature calcination, etching, and chemical bath, to promote the rapid transmission of ions and electrons. Nanofibers, nanobelts, nanotubes, and other diverse structures without adhesive electrode materials can be directly used as electrode materials [23]. Most nanofibers obtained by electrospinning can contribute ideal capacitance due to their directional electron transport and large surface area. In addition, the continuity and flexibility of nanofiber membranes are promising for their applications in adhesive-free electrode materials [24,25].

In this paper, we demonstrate a simple approach to fabricate carbon nanofibers via electrospinning potassium citrate/polyacrylonitrile (PAN) and subsequent calcining processes. The specific surface area and electrochemical properties of the composites can be controlled by varying the potassium citrate amount. Among them, carbon nanofibers with high surface area (347.75 m^2^/g) have shown largely improved energy storage properties, providing a high specific capacitance 404 F/g at current density 1 A/g.

## 2. Materials and Methods

The electrospinning method is modified from our previous reference method [26]. A Bejing Ucalery SS-2535DC electrospinning instrument was used for electrospinning. An N, N-dimethyl formamide (DMF) solution containing 8 wt% PAN was selected for electrospinning. Add potassium citrate with different concentrations (0, 0.25, 0.5, 1, and 1.5 mg/mL, respectively) into the above DMF solution (10 mL) and stir for 12 h. The carbon nanofibers were named C−0, C−0.25, C−0.5, C−1, and C−1.5 and were prepared by different concentrations of potassium citrate (0, 0.25, 0.5, 1, and 1.5 mg/mL). Put the mixture of the precursor solution into a 10 mL syringe, inject it with a metal needle at a speed of 0.1 mm/min (Positive: 15 kV, negative: 3 kV), and then collect the fabric at high pressure on the negative collection plate. The injection thickness is controlled according to the injection time. We use a sample with a thickness of 0.4–0.6 mm, which takes about 6 h to process. 

Next, the samples were dried in a vacuum oven at 65 °C for 12 h, and then the stable fibers were pre-oxidized and carbonized in a nitrogen atmosphere. Preoxidation of the fiber was carried out by heating to 200 °C at 1 °C/min and keeping it for two hours in order to preserve the morphology of the carbon fiber, which was then carbonized at 600 °C (using a heating rate of 5 °C/min). The carbon nanofiber membrane was carefully washed in concentrated HCl for 30 min. The, n the membrane was rinsed with water three times and then immersed in ethanol for 5 min, and the acid-etched nanofiber membrane was dried at 50 °C for 24 h through the vacuum drying oven.

A Hitachi S-4800 field emission scanning electron microscope (FESEM, Hitachi, Tokyo, Japan) was used to observe the morphology of the composite at 15 kV. A Tecnai G2 F30 STWIN field emission transmission electron microscope (HRTEM, FEI, Hillsboro, OR, USA) to observe the micromorphologies of the samples and analyze elemental distribution. The crystal structures of the composites were measured by powder X-ray diffraction (XRD), using a Bruker D8-Advance powder X-ray diffractometer (Bruker, Karlsruhe, Germany) under Cu-Kα radiation at λ = 1.542 Å from 10° to 80° (2*θ*). The Raman spectroscopy test was conducted by an In Via laser Raman spectrometer (Renishaw, Wotton-under-Edge, UK) with an excitation wavelength of 532 nm. X-ray photoelectron spectroscopy (XPS, KRATOS, Manchester, UK) measurement was performed by a KRATOS AXIS-HSi Sigma probe with a monochromatic Al-Kα (1486.6 eV) X-ray source with a constant power of 100 W (15 kV at 10 mA). The surface area was measured by nitrogen adsorption-desorption isotherms using the Brunnauer–Emmett–Teller (BET) method on a Micromeritics ASAP 2020 (Norcross, GA, USA). 

The electrochemical test was performed on a 660E electrochemical workstation (CHI Instruments, Shanghai, China), and the electrolyte used was a 1 M H_2_SO_4_ aqueous solution. In three-electrode systems, 1 mol·L^−1^ H_2_SO_4_ solution was used as electrolyte, a saturated calomel electrode (SCE) served as the reference electrode and a platinum wire worked as the counter electrode. The working electrode was glassy carbon electrode (GCE) and the composite was coated on the electrode in order to test the capacitance from galvanostatic charge/discharge curves (GCD). The specific calculation method follows the following formula [27]:

*C*_S_ = *I*Δ*t*/*m*Δ*E* (1)

where *C*_s_ (F/g) represents the capacitance of the electrode, *I*(A) represents the charge and discharge current, Δ*t*(s) is the discharge time, Δ*E*(V) represents the potential window during the charging process, and *m*(g) indicates the mass of the electrode’s electroactive material.

## 3. Results and Discussion

### 3.1. Characterization of Carbon Nanofibers

**Figure 1:**
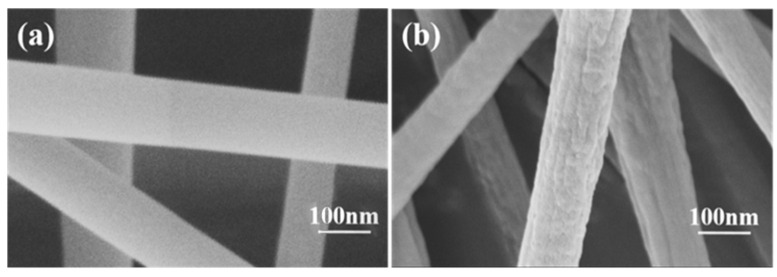
SEM images of (**a**) C−0 nanofiber and (**b**) C−1 nanofiber.

The detailed morphology of carbon fiber membrane based on potassium citrate/PAN after acid etching was detected by SEM. The reason to photograph the material was to discuss the shape changes of the nanofibers after acid etching. Corresponding to C−0, there was no obvious difference in morphology which was obtained after acid etching in Figure 1a. In addition, the nanofibers were not interconnected. It was worth pointing out that the acid etching process had almost no damage to the nanostructure of the carbon fiber, which was very important for applications such as the adsorption of the electrospun fiber [28]. As can be clearly seen in Figure 1b, fiber diameters are about 150 nm with potassium citrate. Moreover, the surface of the product prepared by adding potassium citrate was rough (Figure 1a). 

**Figure 2:**
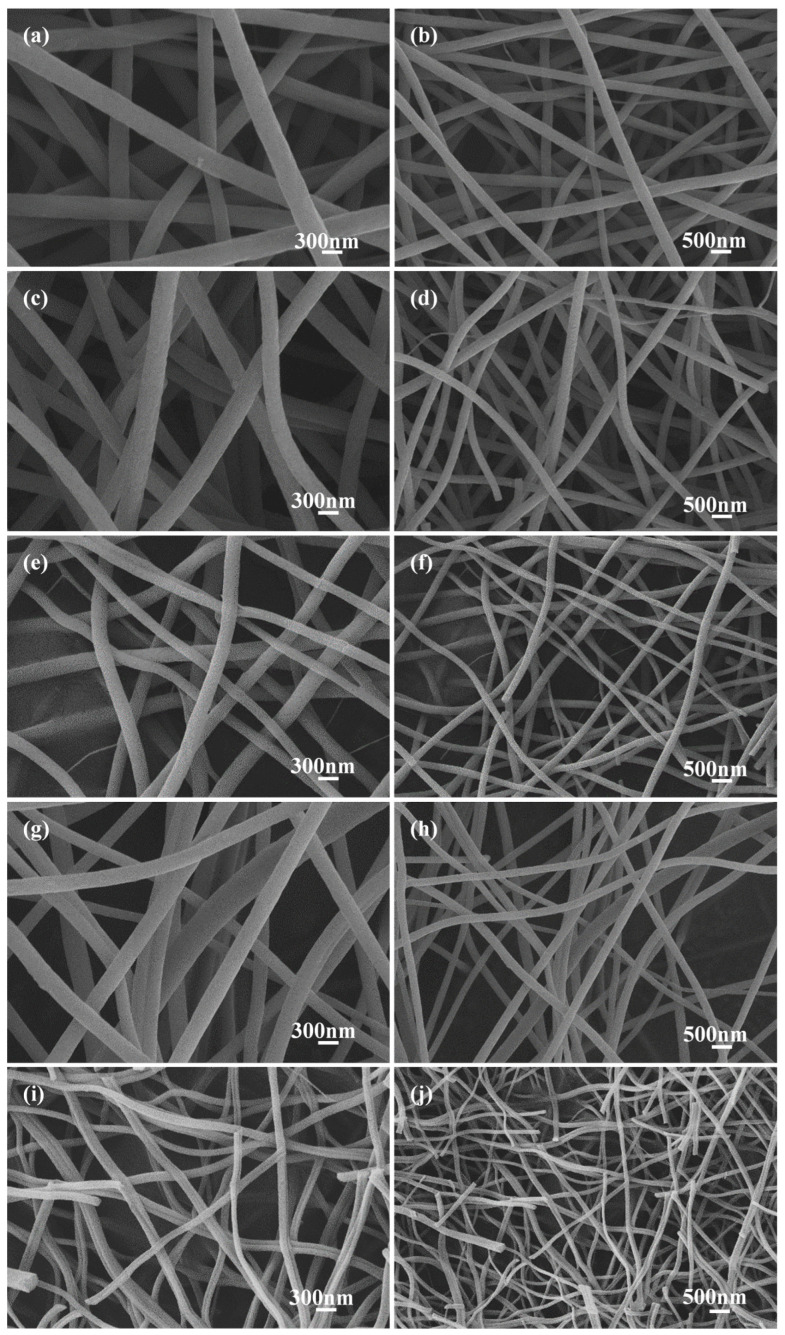
SEM images of carbon fiber membrane based on potassium citrate/PAN: (**a**,**b**) C−0, (**c**,**d**) C−0.25, (**e**,**f**) C−0.5, (**g**,**h**) C−1, (**i**,**j**) C−1.5.

The SEM images of carbon nanofibers (C−0, C−0.25, C−0.5, C−1, and C−1.5) prepared with different potassium citrate concentrations (0, 0.25, 0.5, 1, 1.5 mg/mL) after acid etching are shown in Figure 2. All samples maintained uniform fiber morphology similar to carbon fiber based on PAN. With the increase of the addition amount of potassium citrate, the diameter of the prepared carbon nanofibers became smaller, and the length gradually becomes shorter. It was suggested that the salt addition changed the conductivity of the electrospinning solution, resulting in transformation of the fiber diameter [29]. The fiber average diameter and distributions determined by SEM image analysis (Figure 2) are listed in Figure S1. It was seen that the average diameters of C−0, C−0.25, C−0.5, C−1, and C−1.5 are 290.4 nm, 251.5 nm, 206.8 nm, 186.3 nm, and 113.0 nm, respectively. The obtained fiber (C−1) was thinner than the fiber prepared by pure PAN [28].

Consistent with the SEM results (Figure 2), the diameter of sample C−1.5 had a larger reduction than that of C−0. It was meant that the content of potassium citrate in the spinning precursor solution has a direct effect on the diameter of the product. The rapid shrinkage of the product was caused by the multi-stage decomposition of potassium citrate. The shrinkage was more obvious with the increase of the addition amount of potassium citrate, indicating that the doping had a great influence on the specific surface area of the carbon nanofiber membrane.

Figure S2a shows the TEM image of C−1 nanofiber. The fine defects on the nanofiber surface cannot be hidden with high magnification. Micropores with a diameter of about 1 nanometer are shown in Figure S2b. It was deduced that this is the result of thermal decomposition of potassium citrate on the surface and subsequent acid etching. It was known that the thermal decomposition of potassium citrate includes the following procedures: potassium citrate decomposes into K_2_CO_3_ at high temperature; K_2_CO_3_→CO_2_ + K_2_O; CO_2_ + C→2 CO [30]. Figure S2c–g show C, N, and O elements well dispersed on the nanofibers and cleaned by acid etching. Since carbon fiber was stable in dilute hydrochloric acid solution, the basic morphology of the fiber had not changed. However, after potassium citrate was calcined at high temperature and acid corroded, the surfaces of nanofibers were rougher than that of C−0 nanofiber.

As shown in Table 1, the calculated BET specific surface of C−0 is only 14.93 m^2^/g, while that C−1 reaches 347.75 m^2^/g. The specific surface areas of C−0.25, C−0.5, and C−1.5 are 29.35 m^2^/g, 66.42 m^2^/g, and 74.51 m^2^/g, respectively. The results illustrate that within a suitable concentration range, the specific surface area of carbon nanofibers increases with the increase of potassium citrate. However, when the concentration increased to 1.5 mg/mL, the specific surface area decreased to 74.51 m^2^/g due to the accumulation of excess potassium citrate. The formation of carbon obtained from too much potassium citrate during calcination was accompanied by the release of gas, resulting in the break of carbon nanofiber. However, C−1 had a superior specific surface area, which was attributed to the uniform distribution of potassium citrate in its precursor solution, i.e., potassium citrate is evenly dispersed in the nanofibers through electrospinning. During the further calcination process, the aggregation of potassium citrate was avoided. In the heating process, the multi-stage decomposition of potassium citrate allowed the nanofibers to be influenced by a shrinkage effect, resulting in a significantly smaller aspect ratio of carbon nanofibers. In addition, the potassium atoms and CO_2_ produced during the pyrolysis of potassium citrate created more tiny holes for the carbon nanofibers, increasing the specific surface area, which was beneficial for the improvement of the electrochemical performance.

**Figure 3:**
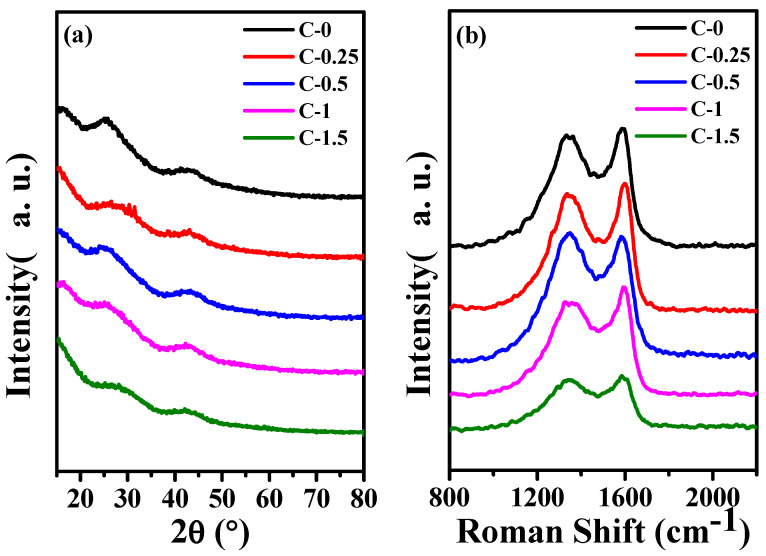
(**a**) The XRD curves of carbon fiber membrane based on potassium citrate/PAN C−0, C−0.25, C−0.5, C−1, C−1.5 and (**b**) The Raman of C−0, C−0.25, C−0.5, C−1, C−1.5.

Figure 3a shows the XRD patterns of C−0, C−0.25, C−0.5, C−1, and C−1.5. All samples illustrated the diffraction peaks of hexagonal graphite (JCPDS, card 41–1487) clearly, which correspond to the (002) and (101) lattice planes of hexagonal graphite at 2θ = 26.4° and 44.4°, respectively [28]. This indicated that all samples belonged to the amorphous carbon structure [31,32]. At the same treatment conditions, the structure of the products obtained from the precursor solutions with different additions of potassium citrate have no obvious difference in the XRD curves.

It can be seen from Figure 3b, C−0, C−0.25, C−0.5, C−1, and C−1.5 all exhibit obvious D (disorder) and G (Graphite) peaks at ≈1350 cm^−1^ and ≈1588 cm^−1^. The R (*I*_D_/*I*_G_) value illustrates the degree of disordered carbon and defective carbon in the composite. The gradual increase in the R value indicates that the carbon nanofiber becomes more and more disordered as the content of potassium citrate increases [33].

**Figure 4:**
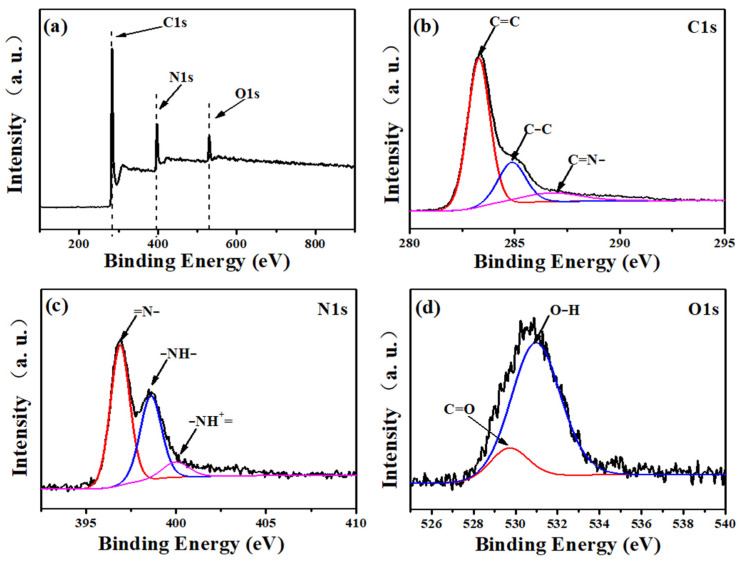
XPS spectra of C−1: (**a**) the full survey spectrum, (**b**) C 1s, (**c**) N 1s, (**d**) O 1s.

The XPS spectrum is used to analyze the surface element valence and chemical composition of carbon fiber membrane based on potassium citrate/PAN. In Figure 4a, the XPS wide-scan spectrum shows the peak of C 1s, with N 1 and O 1 being 283.5, 397.5, and 530.5 eV, respectively. In further analysis, it is shown that the C1s spectrum of Figure 4b reached peaks at 283.3, 284.8, and 287.4 eV, which were attributed to C=C, C-C, and -C=N-, respectively [34]. Furthermore, in the N1s analysis (Figure 4c), the small peak corresponding to -NH- is 398.6 eV. In addition, there are 396.8 eV and 400.0 eV, which are attributed to the characteristic peaks of the two atomic groups =N- and NH^+^=, respectively [35]. Figure 4d showed two low-intensity peaks at 529.7 and 531.0 eV respectively corresponding to C=O and O-H atomic groups on the surface. 

### 3.2. Electrochemical Properties

**Figure 5:**
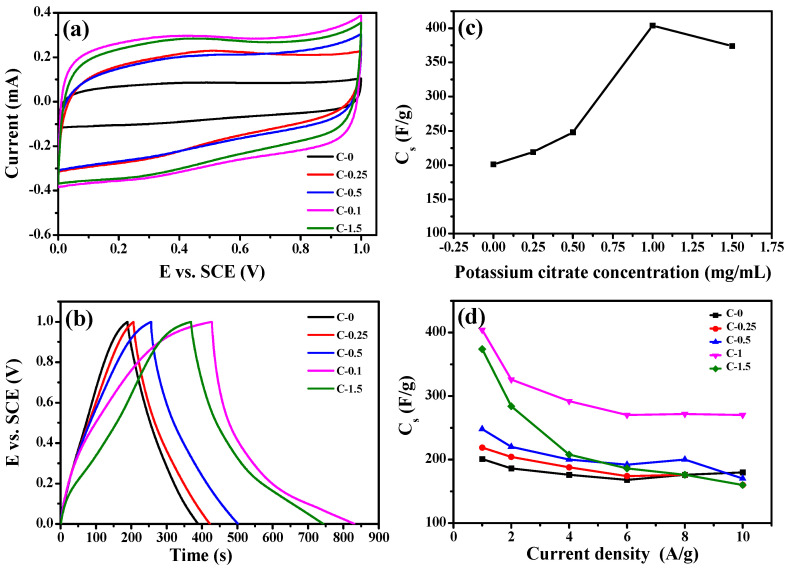
(**a**) The CV curves of C−0,C−0.25,C−0.5,C−1,C−1.5 in 1 mol/L H_2_SO_4_ at 500mv/s at 25 °C, (**b**) The relationship between specific capacitances and composites nanofiber prepared with different potassium citrate concentrations from 0 to 1.5mg/mL at 1.0 A/g, (**c**) Charge-Discharge curves of C−0, C−0.25, C−0.5, C−1, C−1.5 at 1.0 A/g, (**d**) The relationship between thespecific capacitance and the current density of C−0, C−0.25, C−0.5, C−1, C−1.5.

The electrochemical performance of the composites were measured in a three-electrode configuration. Figure 5a displays the cyclic voltammograms of C−0, C−0.25, C−0.5, C−1, and C−1.5 in 1 mol/L H_2_SO_4_ at 25 °C with a scan rate of 500 mV·s^−1^. Obviously, all CV curves were approximately rectangular, which had good symmetry in addition to the largest integral area, indicating that the samples showed a typical double-layer capacitor characteristic. In particular, the cyclic voltammetry performance of the C−1 sample was more prominent compared with other samples. This revealed that C−1 had a large capacitive characteristic and rapid response as an electrode. Figure 5b shows the GCD curves of C−0, C−0.25, C−0.5, C−1, and C−1.5 at a current density of 1 A/g. The curves showed a symmetrical triangle without an obvious IR drop, indicating that excellent reversibility and small internal resistance. In Figure 5c, the capacitances at 1 A/g of C−0, C−0.25, C−0.5, C−1, and C−1.5 are listed as 201, 219, 248, 404, and 374 F/g, respectively. The C−1 composite achieved much higher specific capacitance (404 F/g) compared with other composites. This is higher than that of previously reported carbon nanomaterials (as listed in Table S1). It is worth noting that as the potassium citrate content increases from 0 mg/mL to 1 mg/mL, the capacitance value of the sample had a trend of first rising. However, when potassium citrate content increased to 1.5 mg/mL, the capacitance of C−1.5 decreased to 374 F/g. When the amount of potassium citrate increased to 1.5 mg/mL, the salt exhibited heterogeneous aggregation in the spinning precursor solution, resulting in forming carbon nanofibers with lower specific area compared with C−1. The capacitance retention rates of these composites in 1~10 A/g is shown in Figure 5d. Thus, the specific capacitance of C−1 was still achieved as high as 270 F/g at 10 A/g, and the capacitance retention rate reaches 66.8 %. The specific capacitance of C−0 fiber materials was only 50 % of the specific capacitance of C−1 under 1 A/g. In Figure S3, the Ragone plots of C−1 show that the specific energy was calculated to be much better than the others at the same specific power. It had a good specific energy of 109 W·h·kg^−1^ at a specific power of 980 W·kg^−1^, which was higher than the values of other carbon-based materials [20,36,37]. Furthermore, the specific energy of C−1 remained as high as 73.5 W·h·kg^−1^ even at a specific power of 9800 W·kg^−1^.

## 4. Conclusions

In summary, we have introduced a simple method for designing carbon nanofibers with PAN and potassium citrate by electrospinning, followed by a carbonization process. The addition of potassium citrate increases the specific surface area. The C−1 carbon nanofibers has a high specific capacitance of 404 F/g at 1 A/g, reflecting a high specific surface area utilization. Furthermore, it is hopefully anticipated to be an appropriate electrode material in flexible supercapacitor devices.

## Figures and Tables

**Table 1:** The BET surface area of carbon fiber membrane based on potassium citrate/PAN.

Samples	Surface Area (m^2^/g)
C−0	14.93
C−0.25	29.35
C−0.5	66.42
C−1.0	347.75
C−1.5	74.51

## Data Availability

Not applicable.

## Supplementary Materials

The following supporting information can be downloaded at: https://www.mdpi.com/article/10.3390/membranes12030272/s1, Figure S1: The fiber average diameter and distributions determined by SEM image analysis (Figure 2); Figure S2 C−1 nanofiber of (a,b) HRTEM image, (c) EDS data, (d–g) element mapping; Figure S3: Ragone plots related to specific energy and specific power of C−1; Table S1: Comparison of the specific capacitance of some related materials in the literature.
