# Supplementary material for: Carbon Nanofibers Based on Potassium Citrate/Polyacrylonitrile for Supercapacitors
Source: Membranes (Basel). 2022 Feb 27;12(3):272. doi: 10.3390/membranes12030272 (PMC8951469; doi:10.3390/membranes12030272)
Supplement: Supplementary file 1 [file membranes-12-00272-s001.zip › membranes-1597656-supplementary.pdf]

membranes

Supplementary Materials: Carbon Nanofibers Based on Potassium Citrate/Polyacrylonitrile for Supercapacitors

Wang Zhang [1,2], Ludan Zhang [1], Junqiang Guo [1], Jeongyeon Lee [3], Liwei Lin [2,*] and Guowang Diao [1,*]

[1] School of Chemistry and Chemical Engineering, Yangzhou University, Yangzhou 225009, China; zhangwang@yzu.edu.cn (W.Z.); zhangludan01@163.com (L.Z.); guobyxg@hotmail.com (J.G.)
[2] Department of Applied Bioengineering, Graduate School of Convergence Science and Technology, Seoul National University, Suwon-si 16229, Korea
[3] Institute of Textiles Clothing, Faculty of Applied Science and Textiles, The Hong Kong Polytechnic University, Hung Hom, Hong Kong SAR 999077, China; jaden-jy.lee@polyu.edu.hk
* Correspondence: lin-official@snu.ac.kr (L.L.); gwdiao@yzu.edu.cn (G.D.)

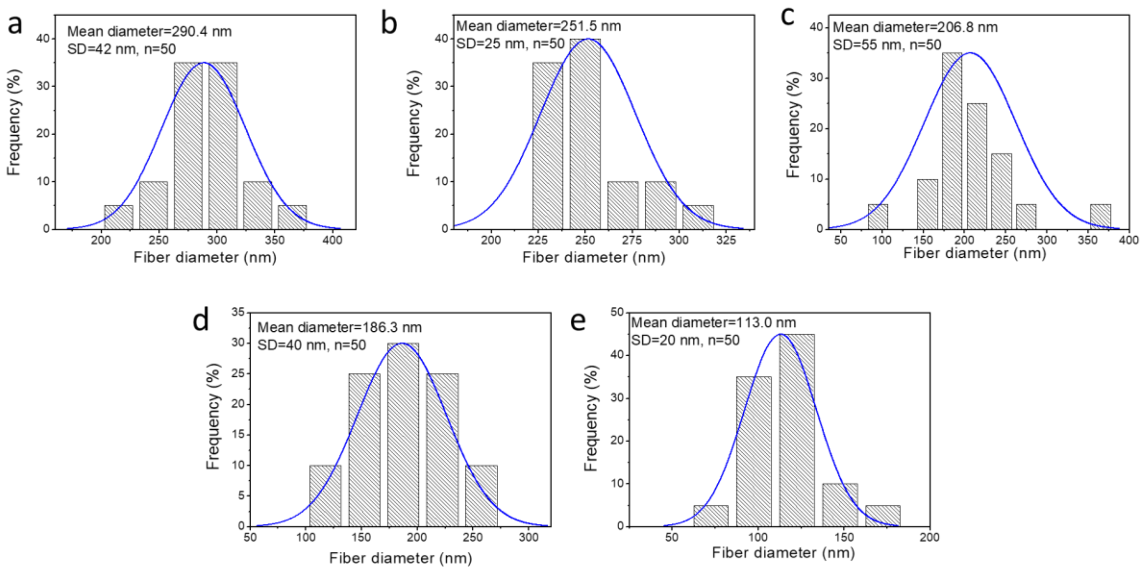

Figure S1. the fiber average diameter and distributions determined by SEM image analysis (Figure 2).

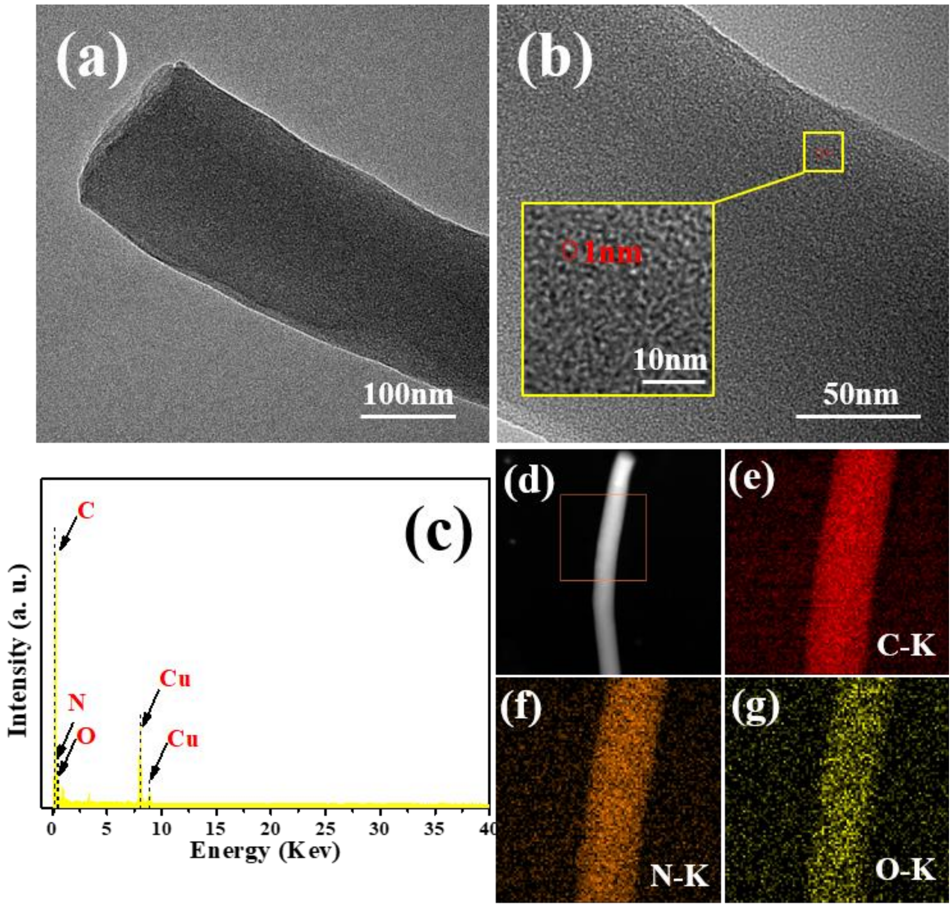

Figure S2. C-1 nanofiber of (a, b) HRTEM image, (c) EDS data, (d-g) element mapping.

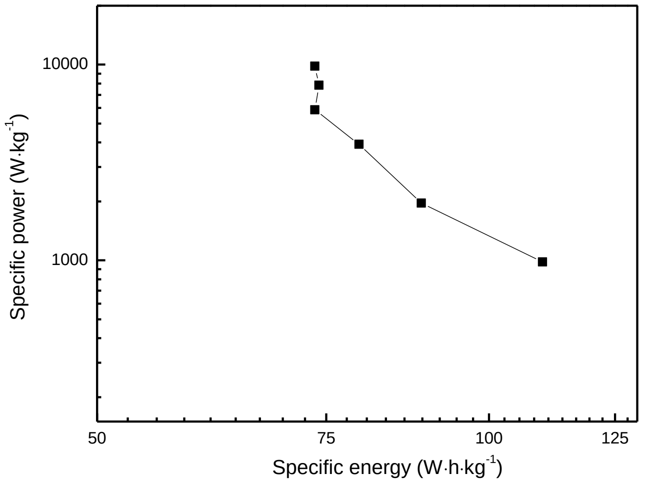

Figure S3. Ragone plots related to specific energy and specific power of C-1.

Table S1. Comparison of the specific capacitance of some related materials in the literature.

Sample	Specific Capacitance ($F \cdot g^{-1}$)	Potential Range (V)	Electrolyte	Current Density ($A \cdot g^{-1}$)	Reference
carbon nanofiber	**404**	0—1.0	1 M H_2SO_4	1.0	our work
N-doped porous carbon nanosheets	350	-1.0—0	6 M KOH	1.0	[1]
porous carbon/carbon cloth	379.5	-1.0—0	6 M KOH	1.0	[2]
activated hierarchical porous carbon	318	-1.0—0	6 M KOH	1.0	[3]
GO/CNT/Pani	294	-0.2—0.8	5 M KOH	0.1	[4]

Reference

[1] D. Kim, X. Jin, Y. Cho, J. Lim, B. Yan, D. Ko, D.K. Kim, Y. Piao, Facile preparation of N-doped porous carbon nanosheets derived from potassium citrate/melamine for high-performance supercapacitors, Journal of Electroanalytical Chemistry, 892 (2021) 115302.

[2] M. Jang, D. Ko, Y. Choi, B. Yan, X. Jin, D.K. Kim, Y. Piao, Self-organized hierarchically porous carbon coated on carbon cloth for high-performance freestanding supercapacitor electrodes, Journal of Electroanalytical Chemistry, 895 (2021) 115456.

[3] K.-d. Seong, X. Jin, D. Kim, J.M. Kim, D. Ko, Y. Cho, M. Hwang, J.-H. Kim, Y. Piao, Ultrafast and scalable microwave-assisted synthesis of activated hierarchical porous carbon for high-performance supercapacitor electrodes, Journal of Electroanalytical Chemistry, 874 (2020) 114464.

[4] E.H. Jo, H.D. Jang, H. Chang, S.K. Kim, J.-H. Choi, C.M. Lee, 3 D Network-Structured Crumpled Graphene/Carbon Nanotube/Polyaniline Composites for Supercapacitors, ChemSusChem, 10 (2017) 2210-2217..
